# Prevalence of oropharyngeal group B Streptococcus colonization in mothers, family, and health care providers

**DOI:** 10.1371/journal.pone.0204617

**Published:** 2018-09-28

**Authors:** Kristina Roloff, Gohar Stepanyan, Guillermo Valenzuela

**Affiliations:** 1 Department of Women’s Health, Arrowhead Regional Medical Center, Colton, California, United States of America; 2 Southern California Permanente Medical Group, Fontana, California, United States of America; Universidade de Lisboa Faculdade de Medicina, PORTUGAL

## Abstract

**Objective:**

To determine the prevalence and serotype of oropharyngeal Group B Streptococcal (GBS) colonization of mothers, their family & friends, and health care providers of recently delivered patients as a potential reservoir of neonatal exposure to GBS.

**Methods:**

This is a prospective, single-center observational study of: (1) patients, (2) their family and friends, and (3) health care providers all of whom may come in close contact with neonates. Oropharyngeal GBS colonization and serotype was determined.

**Results:**

Three hundred and seventy three samples were collected. The prevalence of oropharyngeal GBS colonization among all study participants was 23.1% (N = 86). The most commonly found serotypes were 1b (12.8%, N = 11), III (27.9%, N = 24), and V (17.4%, N = 15). The prevalence of oropharyngeal GBS colonization among mothers was 26% (N = 31/121), 22% (N = 39/178) in family and friends, and 21.6% (N = 16/74) in health care providers.

**Conclusion:**

Group B Streptococcus colonizes the oropharynx in 1 in 5 mothers, family and friends, and health care providers who come in direct contact with neonates. Further research is needed to determine if this potential reservoir for neonatal exposure could lead to early or late onset neonatal GBS colonization or infection.

## Introduction

Implementation of intrapartum antibiotic prophylaxis has significantly decreased the incidence of early onset neonatal Group B Streptococcal (GBS) sepsis in the United States [1–6]. However, the rates of late onset neonatal sepsis have remained largely unchanged and are now comparable to those of early onset disease [7]. The equivalent rate of early and late onset disease seen after intrapartum prophylaxis to rectovaginal GBS carriers suggests an alternative reservoir of GBS that could account for the plateaued rate of early onset, and persistent rate of late onset infections. Case reports and small case series suggest breast milk is a source of exposure in late onset neonatal GBS infection [8–11]. However, only 6% of neonates with GBS infection have mothers with infected breastmilk [12]. Horizontal transmission within neonatal intensive care units has also been described [13–15]. This suggests an alternative reservoir of GBS may contribute to neonatal GBS infection.

Others have described oropharyngeal colonization with GBS [16–23]. However, we were unable to find modern studies that describe the prevalence of oropharyngeal colonization in adults or in persons who come into close contact with neonates. Though the routes and mechanisms of transmission of GBS are poorly known, droplet transmission has been reported [24], and it seems biologically plausible that close facial contact with anyone who is colonized with GBS, such as friends and family members or health care providers could lead to neonatal exposure to this pathogen. Here, we investigate the modern prevalence of oropharyngeal GBS colonization in mothers, along with their family and friends, and health care providers.

## Materials and methods

This is a prospective single-center, cross-sectional, observational study of the prevalence of GBS oropharyngeal colonization of three groups known to come into contact with neonates: (1) mothers, (2) their family and friends, and (3) health care providers.

Group 1 was made of mothers recruited during the postpartum stay, and oropharyngeal samples were collected within 48 hours of delivery. Results of routine 35–37 week rectovaginal GBS cultures were abstracted from participating patients’ charts. These cultures were performed per Center for Disease Control (CDC) guidelines by patients’ OB providers and processed using standard agar plating techniques in the contracted lab. Additional maternal data was abstracted from the medical record at the time of sample collection, and included age, ethnicity, gravidity, parity, gestational age at delivery, date and mode of delivery–vaginal or cesarean delivery.

Group 2 was made of visiting family members and friends. Gender, ethnicity, relationship to the mother of the neonate, and whether the participant was sexually active with the mother was determined for all family or friend study participants prior to collection of the oropharyngeal culture. The participants were asked to answer the following two questions: (1) “Do you routinely wash your hand before holding the baby?” (2) “Do you kiss the baby near the face?”

Group 3 consisted of health care providers, representing a potential nosocomial source. The sample was a convenience sample of any willing health care providers on duty during the study period. Health care providers included registered nurses, respiratory therapists, physician assistants, and physicians directly involved in neonatal care. Our institution has three distinct physical spaces where health care providers are assigned: a 14 bed labor and delivery unit (L&D), 28 bed neonatal intensive care unit (NICU), and 24 bed postpartum unit from which participants were recruited. All health care providers included admitted to regularly come in contact with newborns during their daily clinical practice. We collected their age, gender, title, and primary assigned unit at the time we collected the oropharyngeal swab.

Participants from any group were excluded if they had any upper respiratory tract or oropharyngeal infections in the past 4 weeks, or had used antibiotics within the past 5 weeks prior to their arrival to the hospital. All study participants were blinded to their results.

We collected cultures by swabbing the tonsillar and posterior pharyngeal areas of all participants using the ESwab multiculture collection and transport system. This system contains 1ml of Amies liquid solution to preserve a live culture for up to 48 hours. ESwab multiculture collection and transport system is manufactured by Copan Diagnostics (Murrieta, CA). Studies have shown that ESwab is comparable to Lim Broth enrichment PCR and culture when the bacteria were plated after 24 hours of being in the ESwab’s Amies solution at room temperature [25]. The sensitivity and specificity of ESwab for the detection of GBS within 24–48 hours is 97.2%, and 96.0% respectively. [25] Emeryville Pharmaceuticals (Emeryville, CA) performed culture and serotyping on our specimens. The lab plated all the swabs within 24–48 hours of collection and then used selective Strep B CHROM agar to identify GBS colonies. Upon identification of GBS colonies, the lab transferred the colonies onto additional plates for final confirmation. The isolates that were confirmed to be GBS were further serotyped using Strep-B-Latex latex agglutination assay kit produced by Statens Serum Institut (Denmark) into serotypes Ia, Ib, II, III, IV, V, VI, VII, VIII, and IX.

We performed a descriptive analysis to determine the prevalence and serotypes of oropharyngeal GBS in the study populations. Comparison of demographic characteristics between GBS positive and negative persons was performed using a chi-squared test, two way Student t-test, or Fischer’s exact test where appropriate.

This study was conducted with the approval of the Arrowhead Regional Medical Center (Colton, CA) Institutional Review Board. Written informed consent was obtained from each study participant prior to sample collection. Statistical analysis was conducted with SPSS version 22.0.0.0 (IBM, Armonk, NY, USA). A two-sided p<0.05 was considered statistically significant.

## Results

Three hundred and seventy three samples were collected. Thirty-two percent (N = 121) of samples were obtained from mothers, 48% (N = 178) from family and friends, and 20% (N = 74) from health care providers. Overall, 23.1% (N = 86) cultures demonstrated GBS growth. The most commonly found serotypes were 1b (12.8%, N = 11), III (27.9%, N = 24), and V (17.4%, N = 15). Table 1 shows the serotype distribution of oropharyngeal GBS positive cultures in the three study groups. There were no known cases of early or late onset GBS sepsis among infants of mothers included in this study.

**Table 1:** Serotypes of positive oropharyngeal GBS in all patients, and the three study groups.

Serotype	Group 1: Mothers	Group 2: Family and friends	Group 3: Health care providers	All
Ia	1 (3.2)	3 (7.7)	3 (18.8)	7 (8.14
Ib	4 (12.9)	6 (15.4)	1 (6.3)	11 (12.8)
II	6 (19.4)	3 (7.7)	1 (6.3)	10 (11.6)
III	7 (22.6)	14 (35.9)	3 (18.8)	24 (27.9)
IV	0 (0)	1 (2.6)	1 (6.30	2 (2.3)
V	6 (19.4)	6 (15.4)	3 (18.8)	15 (17.4)
VI	0 (0)	1 (2.6)	1 (6.3)	2 (2.3)
VII	3 (9.7)	1 (2.6)	2 (12.5)	6 (7.0)
VIII	4 (12.9)	1 (2.6)	1 (6.3)	6 (7.0)
IX	0 (0)	3 (7.7)	0 (0.0)	3 (3.5)

Data reported is N (%).

The prevalence of oropharyngeal GBS colonization among mothers was 26% (N = 31/121), 22% (N = 39/178) in family and friends, and 21.6% (N = 16/74) in health care providers.

Demographic data and comparison for oropharyngeal GBS positive and negative study participants are shown for mothers, family and friends, and health care providers in Tables 2–4 respectively. Demographic data were comparable between oropharyngeal GBS positive and GBS negative study participants in the three groups. Ironically, family and friends who were oropharyngeal GBS carriers were more likely to wash their hands prior to holding the baby than those who were not oropharyngeal GBS carriers.

**Table 2:** Demographic characteristics of mothers, compared between oropharyngeal GBS positive and negative study participants.

Demographic characteristics	All heath care providers	GBS Positive–all serotypes	GBS negative	P
N	74	16 (21.6)	58 (78.4)	
Age	42.7±10.5	42.6±10.2	43.0±12.0	0.129
Gender				
Male	3 (4.1)	0 (0)	3 (100)	a
Female	71 (95.9)	16 (22.5)	55 (77.5)	
Unit				
L&D	38 (51.4)	7 (18.4)	31 (81.6)	0.181
NICU	12 (16.2)	1 (8.3)	11 (91.7)	
Postpartum	24 (32.4)	8 (33.3)	16 (66.7)	

Data is mean ± standard deviation, or N (%) where appropriate.

^a^Unable to assess statistically due to low number of males tested.

**Table 3:** Demographic characteristics of mothers, compared between oropharyngeal GBS positive and negative study participants.

Demographic characteristics	Mothers	GBS Positive–all serotypes	GBS Negative	P
N	121	31 (25.6%)	90 (74.4)	
Age	26.2±5.79	26.6±5.75	26.0±5.84	0.614
Gravidity	2.45±1.45	2.32±1.48	2.49±1.48	0.584
Parity	2.06±1.22	1.97±1.25	2.09±1.22	0.635
Ethnicity				
Hispanic	86 (71.1)	24 (77.4)	62 (68.9)	0.582
Caucasian	19 (15.7)	3 (9.7)	16 (17.8)	
Black	14 (11.6)	4 (12.9)	10 (11.1)	
Asian	2 (1.7)	0 (0)	2 (2.2)	
Rectovaginal GBS colonization	8 (6.7)	0 (0)	8 (9.0)	0.084

Assigned unit refers to the primary location that health care provider is assigned to. Data is mean ± standard deviation, or N (%) where appropriate.

**Table 4:** Demographic characteristics of family and friends compared between oropharyngeal GBS positive and negative persons.

Demographic characteristics	Family and friends	GBS Positive–all serotypes	GBS Negative	P
N	178	39 (21.9)	139 (78.1)	
Age	25.84±5.70	25.95±5.40	25.81±5.80	0.890
Gender				
Male	102 (57.3)	23 (60.5)	79 (58.1)	0.787
Female	72 (40.4)	15 (36.5)	57 (41.9)	
Ethnicity				
Hispanic	134 (75.3)	33 (84.6)	101 (72.7)	0.056
Caucasian	20 (11.2)	0 (0)	20 (14.4)	
Black	21 (11.8)	6 (15.4)	15 (10.8)	
Asian	3 (1.7)	0 (0)	3 (2.2)	
Relationship to patient				
Sexual partner	94 (52.8)	22 (57.9)	72 (52.9)	0.536
Friend	34 (20.8)	8 (21.1)	29 (21.3)	
Parent	26 (14.6)	5 (13.2)	21 (15.4)	
Sibling	13 (7.3)	3 (7.9)	10 (7.4)	
Child	4 (2.2)	0 (0)	4 (2.9)	
Patient / Family / Friends (N = 295, 4 cases with missing data)				
Admits routine hand washing before holding baby	247 (82.6)	64 (94.1)	183 (80.6)	0.008
Admits kissing baby on/near mouth	213 (71.2)	46 (67.6)	167 (73.6)	0.516

Data is mean ± standard deviation, or N (%) where appropriate.

One hundred and twenty of the 121 mothers included in the study had documented rectovaginal GBS cultures collected prior to delivery as per standard care (i.e. rectovaginal swab at 35–37 weeks), one patient had unknown rectovaginal colonization. Eight of the 120 (6.7%) showed rectovaginal GBS positive colonization, none of whom had oropharyngeal GBS colonization–though this may have been due to intrapartum exposure to antibiotic prophylaxis. Of the 112 patients who had negative 35–37 week rectovaginal GBS cultures, 31 (27.7%) were oropharyngeal GBS carriers.

A sub-analysis of family members showed no correlation between oropharyngeal GBS colonization, and rectovaginal colonization of the mother (P = 0.206). Of the 26 family members visiting mothers with GBS rectovaginal colonization, only 3 (11.5%) had oropharyngeal GBS colonization, whereas 36 (23.7%) of family and friends who visited mothers with negative GBS rectovaginal cultures (total N = 152) had oropharyngeal GBS isolated.

## Discussion

Oropharyngeal GBS colonization may be an important reservoir contributing to late onset neonatal GBS colonization or infection. Contact with the neonate (e.g. kissing the baby, leaning close to provide resuscitative measures, etc.…) may directly expose the neonate to GBS. Here, we have shown that more than 1 in 5 mothers, family members and friends who visit the neonate, and health care providers are oropharyngeal GBS carriers.

There are few studies that quantify oropharyngeal GBS colonization, but none in the specific population we studied. In 1978 Christensen found that 7% of asymptomatic women in the outpatient gynecology setting had oropharyngeal GBS colonization [16]. Hammerschlag et al. (in 1977) described up to 15% chance of pharyngeal colonization in young healthy girls aged 2–11, and only 5% in girls over 11 years of age [17]. In 1979, GBS was found to be present in 4.4% patients with active pharyngitis [18]. In another study that evaluated diabetic adolescents, only 4.4% of girls and 7.0% of boys had asymptomatic oropharyngeal GBS colonization [19]. In the same study, oropharyngeal GBS was only observed in 1 out of 37 (2.7%) of the control group adolescents [19]. A decade later, Hoffman et al showed the prevalence of oropharyngeal Streptococcus groups B, C, and G combined was 7.2% in asymptomatic adult patients in the outpatient setting [20]. A more recent study of non-pregnant women living in a college dorm showed approximately 20% oral colonization [21]. The prevalence of oropharyngeal GBS that we found in our study population (23.1%) is significantly higher than described in symptomatic and asymptomatic patients in most published studies, though admittedly in different study populations. There could be many possible explanations for the higher prevalence seen in this study, from improved sensitivity and technique of the cultures, to a change in behavior in newer generations.

The majority of early and late onset neonatal GBS infection is caused by GBS serotypes 1a, 1b, and III [26–29]. The most prevalent serotype we found in our study was type III.

Due to their poor immune system at birth, neonates are susceptible to contracting GBS through any of the membranes in the respiratory, gastrointestinal, or genitourinary tract [30]. But there is limited and variable data on mechanism of transmission, which may differ by GBS capsular type [31]. In this study, we have demonstrated that a biologically plausible GBS reservoir exists in the oropharyngeal mucosa of mothers, family and friends, and health care providers that come into contact with newborn neonates. Further study is needed to determine linkage with neonatal colonization and/or infection.

A limitation of this study is that we did not collect neonatal samples, as we did not know if oropharyngeal colonization would be prevalent enough to be a plausible reservoir of neonatal GBS exposure. Also, rectovaginal GBS cultures were collected at the recommended 35–37 weeks of gestation, and not repeated at the time of delivery. Thus, only limited conclusions can be made in regards to the correlation between oropharyngeal and rectovaginal colonization.

## Conclusion

The incidence of early onset neonatal GBS infection has declined since the introduction of screening and treatment guidelines [1–6]. Despite these efforts, early onset neonatal infection has plateaued and late onset neonatal GBS infection rate has remained largely unchanged [6,7]. In this study, we believe we may have identified an important reservoir of neonatal GBS exposure: asymptomatic oropharyngeal colonization of mothers, their family and friends, and health care providers.
